# Stellate Ganglion Block Attenuates LPS-Induced Acute Lung Injury by Activating Sirt3 Regulation of Oxidative Stress and Inflammation

**DOI:** 10.3390/biomedicines12061148

**Published:** 2024-05-22

**Authors:** Shiyun Dai, Jun Ji, Rongrong Li, Lu Gao, Xingying He

**Affiliations:** 1 Department of Anesthesiology, Second Affiliated Hospital of Naval Medical University, Shanghai 200003, China; ldsy0930@163.com (S.D.);; 2 Department of Anesthesiology, Shanghai Children’s Medical Center, School of Medicine, Shanghai Jiao Tong University, Shanghai 200127, China; 3 Department of Anesthesiology, Air Force Medical Center, Air Force Medical University, PLA, Beijing 100142, China; 4 Department of Physiology, Naval Medical University, Shanghai 200433, China; 5 Shanghai Key Laboratory for Assisted Reproduction and Reproductive Genetics, Shanghai 200433, China

**Keywords:** acute lung injury, inflammation, oxidative stress, stellate ganglion block

## Abstract

Stellate ganglion blocks (SGBs) has been applied in clinics to alleviate pain-related syndromes for almost a century. In recent years, it has been reported that SGB can attenuate acute lung injury (ALI) in animals. However, the details of these molecular mechanisms remain complex and unclear. In this study, rats were randomly divided into four groups: group C (receiving no treatment), group NS (receiving the intratracheal instillation of normal saline), group L (receiving the intratracheal instillation of LPS) and group LS (receiving SGB after the intratracheal instillation of LPS). The pathological damage of lung tissue, arterial blood gases, the differentiation of alveolar macrophages (AMs) and inflammatory cytokines (IL-1β, IL-6, IL-10) were detected. Furthermore, the oxidative stress indexes (ROS, CYP-D, T-SOD, Mn-SOD and CAT) in serum and the levels of Sirt3 signaling-associated proteins (JAK2/STAT3, NF-κb p65, CIRP and NLRP3) in the lungs were measured. The results revealed that SGB could attenuate lung tissue damage, improve pulmonary oxygenation, promote the differentiation of AMs to the M2 phenotype, decrease the secretion of IL-1β and IL-6, and increase the secretion of IL-10. Meanwhile, SGB was found to inhibit the production of ROS and CYP-D, and enhance the activities of T-SOD, Mn-SOD and CAT. Furthermore, SGB upregulated Sirt3 and downregulated JAK2/STAT3 and NF-κb p65 phosphorylation, CIRP and NLRP3. Our work revealed that SGB could attenuate LPS-induced ALI by activating the Sirt3-mediated regulation of oxidative stress and pulmonary inflammation; this may shed new light upon the protection of SGB and provide a novel prophylactic strategy for LPS-induced ALI.

## 1. Introduction

Acute lung injury (ALI) is a type of acute and progressive hypoxic respiratory disorder that is caused by various direct or indirect pathogenic factors. ALI is characterized by the extensive infiltration of inflammatory cells, the release of pro-inflammatory mediators, and the occurrence of damage to pulmonary epithelial and endothelial cells, which contributes to pulmonary edema, hypoxemia, and respiratory failure [1]. Despite advances in our understanding of the pathophysiology of ALI, the efficacy of treatment is limited and the mortality rate of ALI remains high.

The stellate ganglion (SG), also known as the cervicothoracic ganglion, constitutes a fusion of the inferior cervical and first thoracic ganglia of the sympathetic trunk [2]. Stellate ganglion block (SGB) is achieved via the injection of local anesthetics such as lidocaine or ropivacaine around the SG, and it is commonly used in the treatment of pain and pain-related disorders [3]. SGB can also be performed as an adjunct treatment for post-traumatic stress disorder (PTSD) [4]. In recent years, studies have revealed that SGB could attenuate ALI via the inhibition of proinflammatory cytokines [5,6,7]. However, the details of these mechanisms remain complex and unclear, prompting us to further explore the mechanisms associated with SGB and implicated in the protection of the lung.

Sirtuins are histone deacetylases that hydrolyze oxidized nicotinamide adenine dinucleotide (NAD^+^), and are associated with pathologies such as inflammation and metabolic diseases [8]. Sirtuins 3 (Sirt3) is a major member of the Sirtuins family that is localized in the mitochondria. An increasing quantity of evidence has indicated that Sirt3 exerts biological functions that are implicated in inflammatory and oxidative stress [9]. Recent study demonstrated that Sirt3 could provide protection against ALI by modulating cellular bioenergetics, alleviating inflammatory responses, and controlling the severity of lung injury [10,11]. As a classical inducer of inflammation, LPS challenge could alter the normal dynamics of both oxidative stress and the inflammatory reaction simultaneously. In addition, after LPS stimulation, the intracellular Cold-Inducible RNA-binding Protein (iCIRP), which is an RNA chaperone protein, can be released into the extracellular space; this recognizes Toll-like receptor-4 (TLR-4) and acts as a damage-associated molecular pattern (DAMP) to promote inflammation and tissue damage [12,13]. The formation of inflammasomes is another cellular mechanism that is induced in response to pathogens or DAMPs; for example, the NOD-, LRR- and pyrin domain-containing protein 3 (NLRP3) inflammasome exacerbates inflammation in a wide variety of diseases [14].

SGB has been found to play a role in lung protection in some scenarios, endowing this classical treatment with new applicative potential. Based on the current literature, this pivotal role seems to be associated with anti-sympathetic and anti-inflammatory effects that inevitably involve Sirt3. Hence, we hypothesized that SGB attenuates ALI via the activation of the Sirt3-mediated regulation of oxidative stress and inflammation.

## 2. Materials and Methods

### 2.1. Chemicals and Reagents

LPS (055: B5) was purchased from Sigma-Aldrich (Merck, Rahway, NJ, USA). PBS and 4% paraformaldehyde were acquired from Beyotime (Shanghai, China). The antibodies used in this study for the immunofluorescence analysis, including anti-CD68, anti-iNOS, anti-CD163, goat anti-mouse Alexa Fluor 488 and goat anti-rabbit Cy3, were all obtained from Servicebio (Wuhan, China). The IL-1β ELISA kit was purchased from Dakewe (Shanghai, China). The IL-10 ELISA kit was purchased from Xitang (Shanghai, China) and the IL-6 ELISA kit was obtained from Thermo Scientific (Vienna, Austria). The ROS Assay Kit and CYP-D Assay Kit were obtained from Xitang (Shanghai, China). The SOD Assay kit and CAT Assay Kit were acquired from Nanjing Jiancheng Bioengineering Institute (Nanjing, China). The antibodies used in the western blot analysis included anti-SIRT3 (CST, Danvers, MA, USA), anti-CIRP (Proteintech, Chicago, IL, USA), anti-NLRP3 (NOVUS, Centennial, CO, USA), anti-NF-κb p65 (CST, Danvers, MA, USA), anti-JAK2 (CST, Danvers, MA, USA), anti-phospho-JAK2 (Y1007/1008) (CST, Danvers, MA, USA), anti-STAT3 (CST, Danvers, MA, USA), and anti-phospho-STAT3 (Tyr705) (CST, Danvers, MA, USA).

### 2.2. Animals and Experimental Design

Adult male Sprague Dawley rats, weighing 200–300 g, were purchased from the Animal Experiment Center of the Naval Medical University (Shanghai, China). All rats were housed for one week to acclimate to their new environment, which comprised a 12 h light/dark cycle and 60 humidity at 22 ± 2 °C. Food and water were supplied ad libitum. All animal experiments were reviewed and approved by the Medical Ethics Committee, Second Affiliated Hospital of Naval Medical University.

All rats were randomly assigned into four groups: group C (receiving no treatment), group NS (receiving the intratracheal instillation of normal saline, 0.5 mL/kg), group L (receiving the intratracheal instillation of LPS, 5 mg/kg) [15], and group LS (receiving SGB half an hour after the intratracheal instillation of LPS).

### 2.3. Establishment of ALI Rats Model

The rats were anaesthetized with 2% isoflurane for approximately 3 min prior to endotracheal intubation. The concentration of LPS was adjusted to 10 mg/mL with normal saline, and ALI was induced via the intratracheal instillation of LPS (5 mg/kg) using endotracheal intubation.

### 2.4. Implement of SGB

In this study, right SGB was performed according to the lateral percutaneous approach, as described by Gulcu N et al. [16]. Once the ALI model had been successfully established, the rat was placed in the left lateral decubitus position. The first and third fingers of the left hand were used to fix the cervical spine of the rat, with the second finger touching C7 at the same time. After the tip of the insulin syringe contacted the surface of the C7 vertebra body, the local anesthetics (0.3 mL of 0.5% ropivacaine) were injected after negative aspiration. Once the rats had recovered from the anesthesia, successful SGB was indicated when the typical signs of Horner syndrome appeared. Rats that did not exhibit Horner syndrome were excluded from this experiment.

### 2.5. Lung Histopathology

The rats were sacrificed, and the middle lobe of their right lung was collected and placed in 4% paraformaldehyde overnight. Then, the fixed tissue was dehydrated, embedded in paraffin, sliced into 4 μm thick sections, and stained with hematoxylin and eosin (H&E) according to the standard protocol. Histological changes in the tissue sections were observed using conventional light microscopy (200 × magnification). Lung injury was evaluated based on the thickening of the alveolar walls, the infiltration of inflammatory cells, pulmonary edema, perivascular edema, and airway injury; this evaluation was performed by two pathologists who were blind to this study. Each criterion was scored as described previously [15].

### 2.6. Lung Wet/Dry (W/D) Weight Ratio

The lung W/D weight ratios of the superior lobes at 24 h and 48 h after the lung injury were calculated to evaluate pulmonary edema. After the blood on the surface of the tissue was removed, the wet weight (W) of the superior lobes of the right lung was recorded. Then, the tissue was dried in an oven at 80 °C for 24 h, 48 h and weighed to obtain the dry weight (D). Pulmonary edema was assessed by calculating the lung wet/dry (W/D) ratio.

### 2.7. Arterial Blood Gas Analysis

At the T0, T1, T2, and T3 time points (0 h, 6 h,12 h and 24 h after different interventions) [6], the rats were anesthetized, and one milliliter of abdominal aorta blood was collected for a blood gas analysis. All samples were analyzed for pH, PaO_2_, PaCO_2_ and SpO_2_ (%) using a blood gas analyzer (i-STAT300, USA) immediately after collection.

### 2.8. Enzyme-Linked Immunosorbent Assay (ELISA)

The animal serum at the T0, T1, T2, and T3 time points was used to analyze the contents of IL-1β, IL-6 and IL-10 using ELISA kits for rats, according to the manufacturer’s instructions.

### 2.9. Oxidative and Antioxidant Indexes Assay

The oxidative indexes (ROS and CYP-D) of the serum were measured at the T0, T1, T2, and T3 time points, according to the manufacturer’s instructions. The antioxidative indexes (total superoxide dismutase/T-SOD, Mn-SOD and CAT activities) were also tested at the different time points mentioned above.

### 2.10. Immunofluorescence Analysis

Alveolar macrophages (AMs) can be divided into the classical activation phenotype (M1) and the alternative activation phenotype (M2), depending on the specific environment; these macrophages express different surface makers and secrete different cytokines [17]. It has been reported that the inducible nitric oxide synthase (iNOS) and cluster of differentiation 163 (CD163) are specific markers for the identification of the M1 and M2 phenotypes, respectively [18,19]. Lung sections were stained with the primary antibodies CD68, iNOS and CD163 overnight at 4 °C, and incubated with the secondary antibodies Alexa Fluor 488-conjugated anti-mouse antibody and Cy3-conjugated anti-rabbit antibody at room temperature for 1 h in the dark; this was after being permeabilized with 0.1% Triton X-100 and blocked with 3% bovine serum albumin (BSA) in PBS. Images were captured using fluorescent microscopy (Nikon Eclipse C1, Nikon) and analyzed with Image J v1.52 software.

### 2.11. Western Blot

The lung tissue was lysed using the radio immunoprecipitation assay (RIPA) lysis buffer. Equal amounts of protein were loaded per well onto a 10% sodium dodecyl sulfate polyacrylamide gel and wet transferred to a Nitrocellulose membrane. Next, the membrane was blocked with 10% BSA in TBST buffer for 1 h at room temperature. After being washed three times with TBST, the membrane was incubated with the primary antibodies, which included anti-SIRT3, anti-CIRP, anti-NLRP3, anti-JAK2 and anti-STAT3; this was performed at 4 °C overnight and was followed by 1 h of incubation with an appropriate IRDye-conjugated secondary antibody at room temperature. After washing, color development was performed using Li-COR ODYSSEY CLx. The relative expression levels of each protein were calculated using Image J v1.52 software.

### 2.12. Statistical Analysis

The statistical analyses were performed using GraphPad software (GraphPad Prism 9.1.0, USA). All data were expressed as mean ± SD. Differences among groups were subjected to analysis of variance (ANOVA). For the comparison of multiple groups, a one-way analysis of variance followed by Tukey’s multiple comparisons test was performed. For the data of different groups at different time points, a two-way ANOVA with Tukey’s multiple comparisons test was performed. *p* < 0. 05 was considered statistically significant.

## 3. Results

### 3.1. Horner Sign Presented after SGB

**Figure 1:**
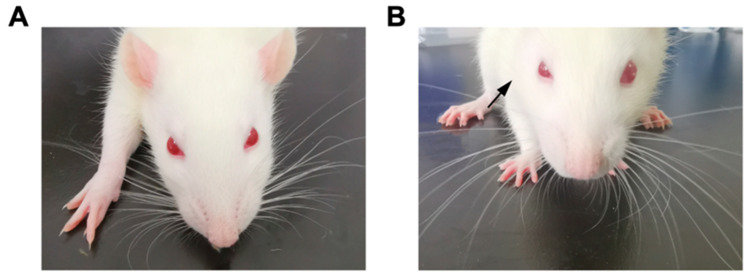
Presentation of signs of Horner syndrome after SGB. (**A**) The rat presented no signs of Horner syndrome without SGB. (**B**) The rat presented ipsilateral signs of Horner syndrome (black arrow) after SGB.

There were no obvious differences between the right and left eyes of the rats that did not receive the SGB treatment (Figure 1A). However, approximately two minutes after SGB was successfully performed, the rats presented signs of Horner syndrome, i.e., ptosis, miosis, enophthalmos and conjunctival congestion on the experimental side (Figure 1B); this continued for approximately 2 h.

### 3.2. SGB Protected Rats from LPS-Induced ALI

**Figure 2:**
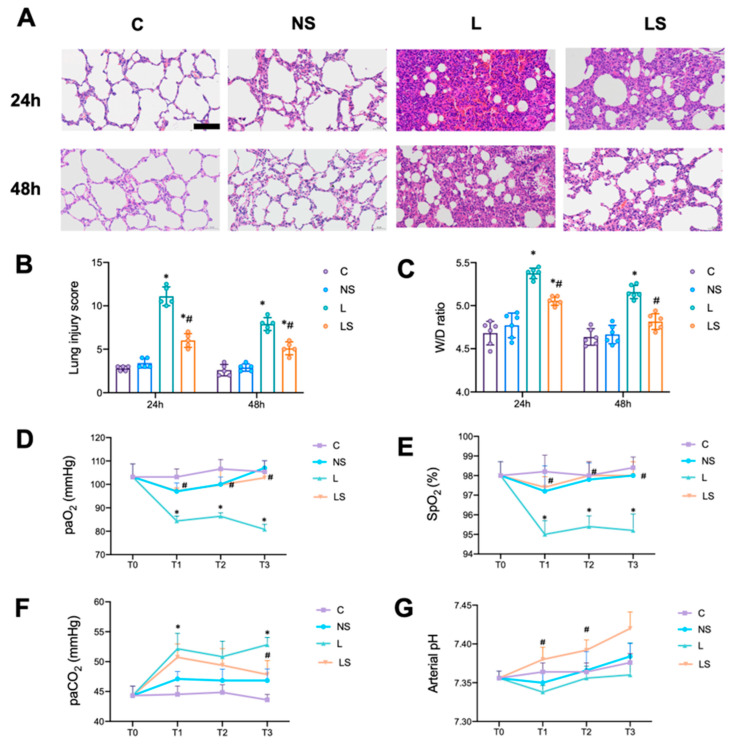
SGB protected rats from LPS-induced ALI. (**A**) Representative images of H&E-stained lung tissue. Scale bar = 100 μm. (**B**) Histopathological scores (Mikawa’s score) of the lung. *n* = 5 per group, * *p* < 0.05 vs. group NS; ^#^ *p* < 0.05 vs. group L. (**C**) The W/D ratio of the lung. *n* = 6 per group, * *p* < 0.05 vs. group NS; ^#^ *p* < 0.05 vs. group L. (**D**–**G**) Comparison of PaO_2_, PaCO_2_, SpO_2_ and arterial pH at the T0, T1, T2, and T3 time points (0 h, 6 h, 12 h and 24 h after different interventions). A two-way ANOVA with Tukey’s multiple comparisons test was performed. *n* = 5 per group, * *p* < 0.05 vs. group NS; ^#^ *p* < 0.05 vs. group L.

Histological changes and the gas present in arterial blood were examined. H&E staining showed the infiltration of inflammatory cells, interstitial edema, the thickening of the alveolar wall and the congestion of the pulmonary capillary 24 h and 48 h after LPS challenge (Figure 2A); thus, the quantitative score of the histological injury was significantly increased (Figure 2B). This was accompanied by an increase in the W/D ratio of the lung (Figure 2C). In contrast, SGB attenuated the histopathological damage caused to the lung and resulted in a significant decrease in the quantitative score and W/D ratio of the lung (Figure 2A–C).

In addition, the arterial blood gas analysis showed that paO_2_ and SpO_2_ were significantly decreased in group L at T1, T2 and T3, while paCO_2_ was significantly increased at T1 and T3 (Figure 2D–F). SGB significantly increased pO_2_ and SpO_2_ at T1, T2 and T3, and significantly increased the pH at T1 and T2 (Figure 2D,E,G).

### 3.3. SGB Inhibited Oxidative Stress

**Figure 3:**
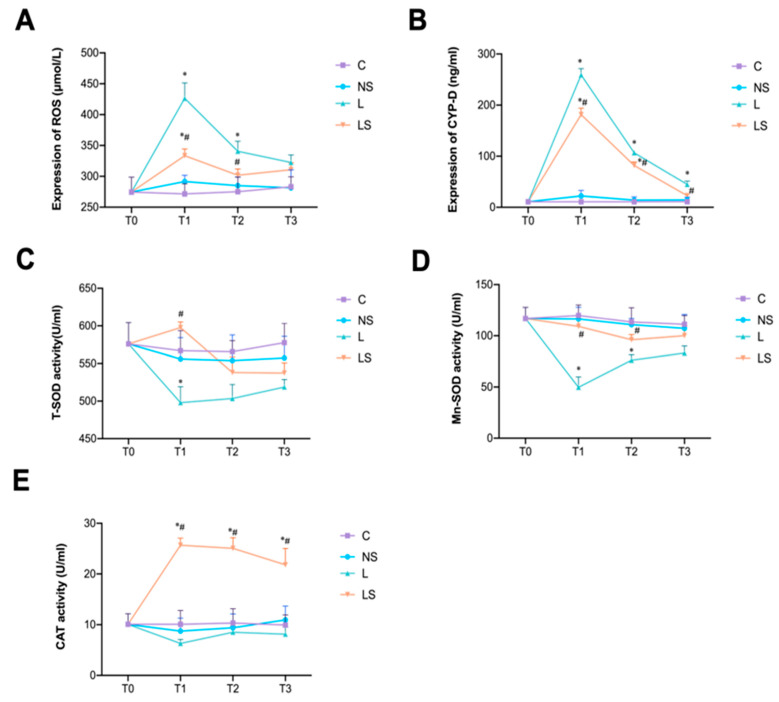
SGB inhibited oxidative stress. (**A**,**B**) Comparison of ROS and CYP-D expression at the T0, T1, T2, and T3 time points (0 h, 6 h,12 h and 24 h after different interventions). *n* = 4 per group, * *p* < 0.05 vs. group NS, ^#^ *p* < 0.05 vs. group L. (**C**–**E**) Comparison of T-SOD, Mn-SOD and CAT activities at different time points. A two-way ANOVA with Tukey’s multiple comparisons test was performed. *n* = 4 per group, * *p* < 0.05 vs. group NS, ^#^ *p* < 0.05 vs. group L.

LPS-triggered oxidative stress was accompanied by an increase in ROS and CYP-D, which reached their peak at T1; however, this oxidative stress was significantly decreased by SGB (Figure 3A,B). Meanwhile, the activities of T-SOD and Mn-SOD were inhibited in group L, but reversed by SGB at T1 (Figure 3C,D). The same pattern was observed in the activities of CAT (Figure 3E).

### 3.4. SGB Inhibited M1-AM Differentiation and Pro-Inflammatory Cytokines Secretion

**Figure 4:**
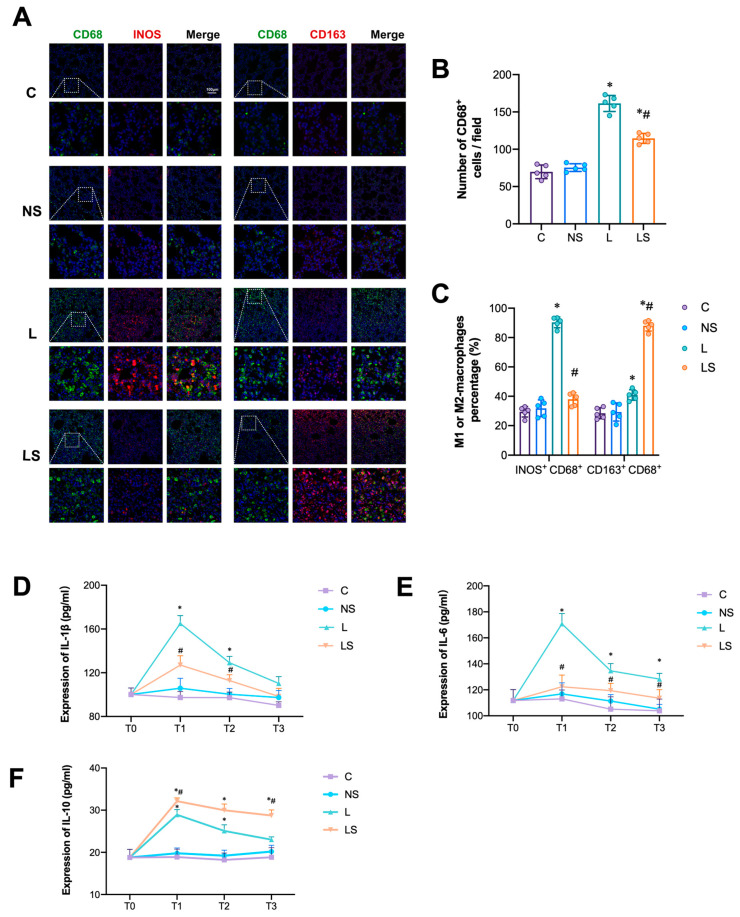
SGB inhibited M1-AM differentiation and the secretion of pro-inflammatory cytokines. (**A**) Immunofluorescence staining of M1-macrophages (CD68^+^/green, iNOS^+^/red) and M2-macrophages (CD68^+^/green, CD163^+^/red). Scale bar = 100 μm. (**B**) Percentage of macrophages. A one-way ANOVA with Tukey’s multiple comparisons test was performed. *n* = 4 per group, * *p* < 0.05 vs. group NS, ^#^ *p* < 0.05 vs. group L. (**C**) Comparison of M1 and M2 macrophages among different groups. A one-way ANOVA with Tukey’s multiple comparisons test was performed. *n* = 4 per group, * *p* < 0.05 vs. group NS, ^#^ *p* < 0.05 vs. group L. (**D**–**F**) IL-1β, IL-6 and IL-10 expression at the T0, T1, T2, and T3 time points (0 h, 6 h, 12 h and 24 h after different interventions). A two-way ANOVA with Tukey’s multiple comparisons test was performed. *n* = 4 per group, * *p* < 0.05 vs. group NS, ^#^ *p* < 0.05 vs. group L.

CD68 is a common marker for active macrophages. Numerous CD68^+^ cells were observed in group L and LS, while there were significantly fewer CD68^+^ cells in group LS than in group L (Figure 4A,B). Macrophages are mainly composed of the M1 and M2 phenotypes; the former act as pro-inflammatory macrophages while the latter act as anti-inflammatory macrophages. In group L, most of the CD68^+^ cells were iNOS-marked M1 macrophages, and the percentage of M1 macrophages was the highest amongst all the groups (Figure 4C). In contrast, most of the CD68^+^ cells after SGB were CD163-marked M2 macrophages, and the percentage of M2 macrophages was significantly higher than that in group L (Figure 4C). Consistent with the above results, SGB significantly decreased the secretion of pro-inflammatory cytokines (IL-1β and IL-6) and increased the level of anti-inflammatory IL-10 compared with group C (Figure 4D–F).

### 3.5. Effects of SGB on Sirt3 Signaling-Associated Proteins

**Figure 5:**
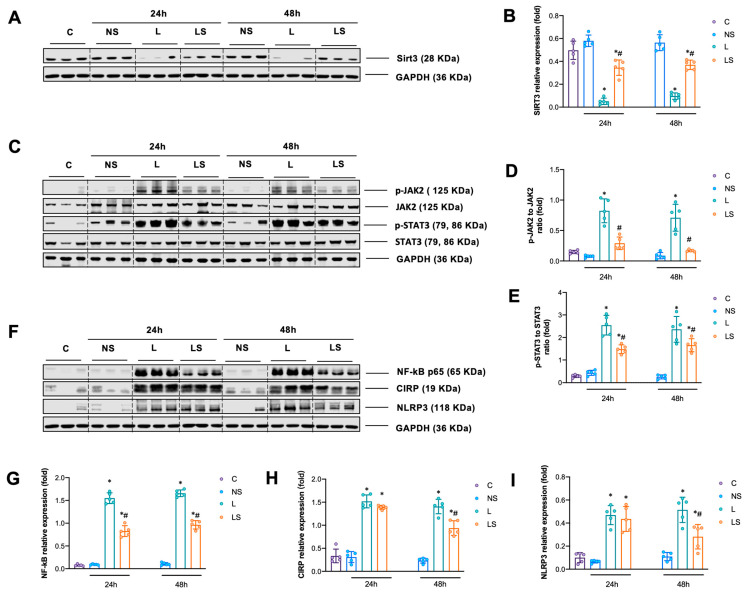
(**A**,**B**) Representative WB images of SIRT3 and relative expression of SIRT3. (**C**–**E**) Representative WB images of p-JAK2, JAK2, p-STAT3, STAT3 and relative expression of p-JAK2/JAK2, p-STAT3/STAT3. (**F**–**I**) Representative WB images of NF-κb p65, CIRP, NLRP3 and relative expression of NF-κb p65, CIRP, NLRP3. A two-way ANOVA with Tukey’s multiple comparisons test was performed. *n* = 5 per group, * *p* < 0.05 vs. group NS, ^#^ *p* < 0.05 vs. group L.

LPS challenge significantly downregulated the expression of Sirt3 expression, while SGB significantly promoted it at 24 h and 48 h (Figure 5A,B). The phosphorylation of JAK2/STAT3 and NF-κb p65 was significantly enhanced after LPS challenge, but SGB significantly inhibited it at 24 h and 48 h (Figure 5C–G). In addition, as a marker for the activation of the TLR-4 inflammatory pathway, the expression of CIRP and NLRP3 was notably elevated at 48 h after LPS challenge, but it obviously decreased after SGB (Figure 5F,H,I).

## 4. Discussion

ALI is a complex pulmonary destructive disease with limited therapeutic approaches. After LPS challenge, the cellular metabolism is reprogrammed and pulmonary inflammation and injury are thus aggravated [20]. SGB has been widely used in clinical practice, with some basic research demonstrating that SGB can attenuate sepsis-induced ALI by decreasing the expression of NF-κb [6] and improve pulmonary function by inhibiting the hyperactivity of the sympathetic nervous system [5]. The above studies suggest that SGB could be employed as a promising alternative in lung protection. Encouragingly, this study confirms that accurate SGB can attenuate LPS-induced ALI in rats and speculates that this protective role is associated with Sirt3.

Sirt3 is a major NAD^+^-dependent sirtuin family member that deacetylates key mitochondrial antioxidant enzymes to reduce ROS levels [21,22]. The targets of Sirt3 include the detoxification of ROS and the regulation of the mitochondrial permeability transition pore (mPTP), which are connected to the cellular metabolic process and protect cells from various stresses [23]. Current studies have confirmed that SIRT3 may effectively ameliorate ALI [10,11]. In our study, LPS challenge resulted in the downregulation of Sirt3, which led to an increase in oxidative stress and dominant M1 macrophages; this was reversed by SGB. Following a decrease in the oxidative stress and ratio of M1/M2 macrophages, the production of oxidants, inflammatory and related cytokines also tended to decrease; thus, the pulmonary inflammation was alleviated to some extent.

As a classical TLR-4 agonist and Pathogen-Associated Molecular Pattern (PAMP), LPS stimulated AMs and caused the subsequent release of iCIRP. CIRP is a stress response protein that is regulated by many stress conditions such as hypoxia, glucose deprivation and H_2_O_2_ [24]. In inflammatory states, the levels are extracellular CIRP (eCIRP) are elevated, mediating tissue injury and inflammation [25,26,27]. In addition, eCIRP has been found to activate NLRP3 in mouse lung vascular endothelial cells (MLVECs), increasing the levels of caspase-1 and IL-1β and inducing pyroptosis [28]. NLRP3, an important component of the innate immune system, forms a pro-caspase-1-activating platform in response to DAMP (CIRP) or PAMP (LPS), followed by the activation of caspase-1 and the generation of the pro-inflammatory cytokine IL-1β/IL-18 [29]. The release of mature IL-1β/IL-18 also indicates the activation of Nlrp3/caspase-1 signaling. Our results showed that SGB could significantly inhibit the LPS-induced upregulation of CIRP and NLRP3, which is involved in NLRP3 inflammasome assembly and pyroptosis. As an inflammatory and programmed mode of cell death, pyroptosis is characteristic of plasma membrane rupture and IL-1β/IL-18 release [30], which would exacerbate acute inflammation in the manner of positive feedback. Although the roles of SGB in pyroptosis are not the concern of this study, they are worthy of further study. At the same time, stimulation by LPS activated the JAK2/STAT3 and NF-κb pathway to amplify the inflammatory response and directly damage lung tissues [31,32], which was inhibited by SGB also. We partly attributed this pivotal role of SGB to Sirt3, due to the potential relationship between STAT3/ NF-κb and mitochondria [33,34,35]. However, research on this crosstalk is scarce, so further research is needed.

**Figure 6:**
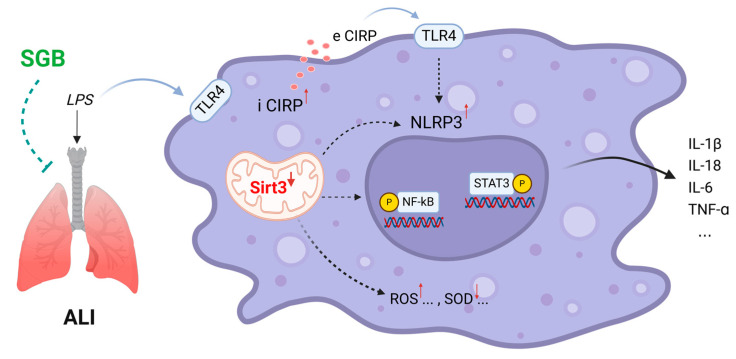
Proposed schematic representation of SGB attenuation LPS-induced ALI. 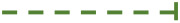 denotes inhibition.

In summary, our results show that SGB can effectively attenuate LPS-induced ALI in rats via the promotion of the Sirt3-mediated regulation of oxidative stress and pulmonary inflammation. The proposed mechanism of SGB lung protection is summarized in Figure 6.

Our study has some limitations. Firstly, we only performed an animal experiment, so how the effects of SGB can be mimicked in vitro should be considered in order to verify the outcomes. Secondly, we did not explore the detailed crosstalk among SIRT3 signaling-associated proteins, so future experiments should focus on the signaling pathway involved. Thirdly, CIRP and Sirt3 are natural innate molecules, so CIRP and Sirt3 knockout animal experiments should be carried out in future research.

## Data Availability

The datasets generated or analyzed during the current study are available from the corresponding author upon reasonable request.

## Abbreviations

SGB: Stellate ganglion block; LPS: Lipopolysaccharide; ALI: Acute lung injury; IL: Interleukin; TNF: Tumor necrosis factor; AMs: alveolar macrophages; ROS: Reactive oxygen species; Mn-SOD: Manganese superoxide dismutase; CAT: Catalase; CYP-D: Cyclophilin D; SIRT3: Sirtuin 3; CIRP: Cold-inducible RNA-binding protein; NLRP3: NOD-, LRR- and pyrin domain-containing protein 3.
